# *In varietate concordia* – cluster analysis of EQ-5D-5L value sets in European Union countries

**DOI:** 10.1007/s11136-024-03872-0

**Published:** 2024-12-12

**Authors:** Vera Pinheiro, Tallys Feldens, Juanita A. Haagsma, João Vasco Santos

**Affiliations:** 1 Public Health Unit, ULS Matosinhos, Matosinhos, Portugal; 2 Centre for Health Technology and Services Research, CINTESIS, Rua Dr. Plácido da Costa, Porto, 4200-450 Portugal; 3 MEDCIDS, Faculty of Medicine, University of Porto, Porto, Portugal; 4 Federal University of Paraná, Curitiba, Brazil; 5 Department of Public Health, Erasmus MC University Medical Center, Rotterdam, The Netherlands; 6 Public Health Unit, ULS Santo António, Porto, Portugal

**Keywords:** EQ-5D-5L, Health economics, Health technology assessment, European Union

## Abstract

**Background:**

The transferability of health policies in the European Union (EU) faces challenges due to economic, political, and healthcare system factors, including cross-country differences in health preferences. In order to aid policymaking, previous research has grouped EU countries based on geographical proximity or literature-based criteria, but not on health preference data. The EQ-5D-5L instrument, which measures health-related quality of life and reflects unique national health preferences shaped by cultural and social factors, is used to evaluate policies, technologies and interventions, but has not been used to group countries. Thus, this study aims to identify clusters of EU-27 countries with similar preference patterns using published EQ-5D-5L value sets.

**Methods:**

Hierarchical agglomerative clustering was employed on 13 EU-27 countries’ EQ-5D-5L value sets, first analyzing regression coefficients for each dimension-level and then creating a simulated coefficient distribution considering uncertainty.

**Findings:**

Five clusters of EU-27 countries emerged: (1) Belgium, Netherlands, Sweden, Germany); (2) Hungary, Italy, Portugal; (3) Poland, Romania; (4) France, Spain; (5) Denmark, Ireland. All clusters except cluster 5 gave higher importance to “Pain and Discomfort”; all clusters except cluster 1 gave less importance to “Usual Activities”. “Anxiety and Depression” and “Pain and Discomfort” had the largest heterogeneity in valuation across severity level.

**Interpretation:**

Identified clusters of countries with distinct health preferences provide insights for health policy transferability in the EU. Recognizing countries with similar health preferences can aid EU policymaking and transferability efforts, particularly in early-stage policymaking, while also fostering closer collaboration on health policy decisions and best practice sharing. Further development of national value sets within the EU is necessary for a more accurate representation.

**Supplementary Information:**

The online version contains supplementary material available at 10.1007/s11136-024-03872-0.

## Abstract (plain language summary)

This study explores health preference patterns across European Union (EU) countries using the EQ-5D-5L tool, which measures health-related quality of life considering five dimensions: mobility, self-care, usual activities, pain/discomfort, anxiety/depression. Unlike previous research that grouped countries by geographical proximity or specific cultural factors, this analysis clusters EU-27 countries based on actual health preference data, creating five distinct groups with similar health priorities. These findings show that health preferences don’t necessarily align with geography, highlighting diverse health priorities across regions. This new perspective can guide EU policymakers in tailoring health policies, treatments, or technologies to better match each cluster’s preferences. By understanding these patterns, EU countries can share strategies and best practices more effectively, supporting better decision-making and closer collaboration on healthcare policies across the region.

**Supplementary Information:**

The online version contains supplementary material available at 10.1007/s11136-024-03872-0.

## Introduction

When it comes to improving health, in particular health-related quality of life (HRQoL) – which can be summarized as the value ascribed to a person’s life for the duration of that life, by the individual or society, as a result of his or her own health and determinants such as personal behavior, medical care, health policy [1–3] - policymakers strive to evaluate the success of health policies, technologies, or interventions across different contexts. This can be challenging due to diverse demographic, economic and healthcare system-related factors, but also because HRQoL is subjective and dependent on values, beliefs, and cultural context [1–3]. The most common way to perform this assessment is through economic evaluations (EE) and, in particular, cost-utility analysis, which often use quality-adjusted life years (QALYs) as outcome measures, a metric that considers both life expectancy and quality of life [4]. Yet, economic evaluations and health technology assessments (HTAs), are typically time-consuming, costly, and require significant expertise. Consequently, it is not feasible to conduct an EE or HTA for every policy, intervention or technology. Health decision makers, however, often have access to previously published EEs or HTAs, though these are frequently from other jurisdictions. This requires assessing if and how results from another jurisdiction can apply locally — a process referred to as transferability, often also called generalizability [1, 2]. Before undertaking a new EE or HTA, it’s essential to first examine available studies from other settings and consider their applicability – an approach that is especially useful for decision-makers facing budget or time constraints. Given the growing demand for evidence-informed decision-making, there is increased interest in evaluating the transferability of EEs and HTAs to adapt external findings for local use. In this context, transferability refers to the extent to which the effects, outcomes, and cost-effectiveness of a health technology, intervention or policy implemented in one setting, population, or context can be generalized, adapted, or applied to another setting, considering differences in population demographics, healthcare systems, and socio-economic factors [1, 2].

Preference-based multi-attribute utility instruments (MAUIs), such as the EQ-5D, measure HRQoL and are preferred for estimating utilities in QALY calculations [5]. The EQ-5D-5L, introduced in 2012, assesses HRQoL by asking respondents to assess their health across five dimensions (mobility, self-care, usual activities, pain/discomfort, anxiety/depression), with five levels of severity (1 - no problems to 5 - extreme problems), defining unique 3,125 health states. A value set derived from a representative sample of the population is then applied to the respondents’ choices in order to determine a utility index for each health profile (0, death, to 1, full health), allowing for the estimation of QALYs [6]. The EuroQol Valuation Technology (EQ-VT) protocol ensures a consistent approach across studies [7]. Since 2012, 31 national value sets for EQ-5D-5L which used EQ-VT have been published. Differences in value sets between countries may reflect true differences in health state preferences or between protocol versions [8, 9]. Identifying groups with similar preference patterns can guide decision-makers in selecting candidate countries for health policy or intervention transferability. As previously stated, conducting full EE or HTAs can be very complex and may not always be feasible. Therefore, especially in early-stage policy or technology implementation decisions in the absence of detailed EEs, finding *a priori* candidate countries may be a practical solution. As health preference patterns are key features related to health policy transferability [1, 2], this study proposes a new way of identifying these countries based on a shared perspective on their population’s health preferences. This could reduce initial costs, offering a pragmatic approach to prioritize or preliminarily assess policies, technologies or interventions before investing in a full evaluation.

In fact, previous research has shown distinct preference patterns across countries, with some studies estimating common currency values for groups with shared characteristics [8–14]. For instance, Roudijk et al. [8] assessed national value sets based on the relative importance of EQ-5D-5L dimensions, the value scale length and the distribution of values over the scale, identifying three groups with distinct preference patterns: Asian, Eastern European and Western countries. Łaszewska et al. [12] used literature-based attributes (culture/religion, linguistics, healthcare system and financing and sociodemographic aspects) to develop 5 groups of English-speaking, Nordic, Central-Western, Southern and Eastern European countries. Still, a key limitation of these studies is the predefined grouping of countries based on literature or *a priori *concepts. As health preferences are crucial for transferability, considering similarities across countries is essential. While cross-country differences exist, the European Union (EU) presents a cohesive sociocultural background and governance structure, making it an ideal region for such an analysis. However, no study has assessed value set differences in the EU in a truly data-driven manner, including through cluster analysis, the most widely used data segmentation method [15].Thus, this paper aims to identify clusters of countries with similar preference patterns based on published EQ-5D-5L value sets in the EU-27.

## Methods

### Included studies

As of March 1st 2023, we included 13 EQ-5D-5L value sets from EU-27 countries from the EuroQol website [16] — Belgium [17], Denmark [18], France [19], Germany [20], Hungary [21], Ireland [22], Italy [23], Netherlands [24], Poland [25], Portugal [26], Romania [27], Spain [28] and Sweden [29]. To the best of our knowledge, no new EU-27 national value set has been published as of July 2024. EQ-5D-5L value sets were considered for the analysis as they are more sensitive and precise and have specific methodological protocols in comparison with EQ-5D-3L value sets. Firstly, a descriptive analysis of the value sets was perfomed. Secondly, the cluster analysis was performed.

### Comparison of EQ-5D-5L value sets

Firstly, a summary descriptive analysis of the included EQ-5D-5L value sets was performed, comparing EQ-VT protocol version, number of respondents, preference elicitation techniques, model type, and publication year for each value set. Similarly to Roudijk et al. (2022) [8], we then performed a comparison of (1) the relative importance of each dimension in each value set and (2), in particular, the relative importance of the functional dimensions (i.e., mobility (MO), self-care (SC), and usual activities (UA)) vs. symptomatic dimensions (i.e., pain/discomfort (PD), anxiety/depression (AD)) for all countries; (3) coefficient range across dimensions and countries; and lastly, (4) severity level 5 coefficients across dimensions and countries.

### Cluster analysis of EQ-5D-5L value sets

Secondly, a cluster analysis was conducted on the final regression model coefficients and standard errors of the value sets in two consecutive stages: firstly, using only the final regression coefficients and, secondly, using a simulated dataset (obtained using the coefficients and standard errors) to account for modeling uncertainty. In our dataset, each value set had 25 regression coefficients (including a reference level) and respective standard errors, with each coefficient representing the estimated utility for each level and each dimension compared to the reference level (lowest severity). Clustering was performed through a hierarchical agglomerative nesting method (Agnes). Using Agnes, each observation (i.e., coefficient for each level and each dimension, for every country) starts with its own cluster. They are then iteratively combined based on the smaller Euclidean distance of the values between them. In this study, for two given countries, the closer the values of their coefficients in each level and dimension, the higher the chance these two countries will end up nested in the same cluster. On each iteration, the nesting process builds a hierarchy, forming dendrograms which represent the relative closeness of the observations among the formed clusters. This strategy does not rely on a predefined number of groups (unlike K-means clustering) and was chosen due to its data-driven approach and relative easiness of interpretability [15]. Single, complete, average, and Ward linkage algorithms were tested, and agglomerative coefficients (AC) were calculated (which measure the strength of the clustering structure, with values ranging from 0 to 1 and higher values indicating a more well-defined clustering structure) to determine the most suitable method. Cluster dendrograms were then evaluated with the Elbow method to identify the optimal number of clusters (which plots the explained variance as a function of the number of clusters). Finally, we computed the Dunn Index to assess separation and compactness of the clusters, another aspect of good quality clustering (through the measurement of the ratio between the smallest inter-cluster distance – separation - and the largest intra-cluster distance – compactness, with higher values indicating better clustering). In the first-stage analysis, the coefficients for each level and each dimension of the value set were used. In the second stage analysis we used a simulated dataset obtained through the replication of the distribution of each coefficient for every country with bootstrapping with random sampling, using the given coefficients and standard errors. Sensitivity analysis using K-means clustering was also performed and yielded identical results.

Based on Poudel et al. [10], who found statistically significant differences between specific EQ-5D-5L dimension levels regarding EQ-VT protocol version across studies, the EQ-VT protocol version for each value set was then used to perform analysis in two groups: (1) the 11 value sets that used EQ-VT protocol version 2.0 or 2.1 – considered the most robust; and (2) the total 13 value sets. Finally, we performed an assessment of the relative importance of each EQ-5D-5L dimension in each cluster, based on the comparison of values for health states with single-dimension level 5 issues (51111, 15111, 11511, 11151, 11115) and single-dimension level 3 issues (31111, 13111, 11311, 11131, 11113) in all clusters, both in the 11 and 13 country analyses and in both the coefficient and simulated analyses.

All analyses were conducted using R software version 4.0.1.

## Results

### Comparison of EQ-5D-5L value sets

In total, 13 value sets have been published for EU-27 countries, 31% in the Western European region (according to the United Nations Statistics Division (UNSD)), 23% in the Northern European region, Southern European region and Eastern European region – Table 1. Most (85%) used EQ-VT protocol versions 2.0 and 2.1, while only the Netherlands and Spain (15%) used version 1.0. Year of publication ranged from 2016 to 2023. 54% of studies used stratified sampling. Regarding elicitation techniques, 77% of studies used a hybrid final model, combining both cTTO and DCE data. Some studies (15%) used a Censored Tobit model with TTO data only, while others opted for different models. The number of respondents varied, with some falling below the recommended threshold of 1000 for deriving utilities from EQ-5D-5L [30].

**Table 1:** Summary information on included EQ-5D-5L study characteristics: country, UNSD sub-region, publication year, sampling method, sample size, protocol version, preference elicitation technique(s), value set model and ranking of relative importance of dimensions in each value set

Country	UNSD sub-region	Publication year	Sampling method	Sample size	Protocol version	Preference elicitation technique(s)	Value set Model	Ranking
Belgium	Western Europe	2022	stratified sampling	892	EQ-VT 2.1	cTTO + DCE	Hybrid	PD > AD > MO > UA > SC
Denmark	Northern Europe	2021	random sampling	1014	EQ-VT 2.1	cTTO + DCE	Hybrid	AD > PD > MO > SC > UA
France	Western Europe	2020	quota sampling	1000	EQ-VT 2.0	cTTO + DCE	Hybrid	PD > MO > SC > AD > UA
Germany	Western Europe	2018	quota sampling	1158	EQ-VT 2.0	cTTO + DCE	Hybrid	PD > AD > SC > MO > UA
Hungary	Eastern Europe	2020	quota sampling	1000	EQ-VT 2.1	cTTO + DCE	Tobit (cTTO)	MO > PD > SC > AD > UA
Ireland	Northern Europe	2018	stratified sampling	1000	EQ-VT 2.0	cTTO + DCE	Hybrid	AD > PD > MO > SC > UA
Italy	Southern Europe	2021	stratified sampling	1182	EQ-VT 2.1	cTTO + DCE	Hybrid	PD > MO > AD > SC > UA
Netherlands	Western Europe	2016	stratified sampling	979	EQ-VT 1.0	cTTO + DCE	Tobit (cTTO)	AD > PD > MO > UA > SC
Poland	Eastern Europe	2019	quota sampling	1252	EQ-VT 2.0	cTTO + DCE	Hybrid	PD > MO > SC > AD > UA
Portugal	Southern Europe	2019	stratified sampling	1000	EQ-VT 2.0	cTTO + DCE	Hybrid	PD > MO > SC > AD > UA
Romania	Eastern Europe	2019	stratified sampling	1493	EQ-VT 2.1	cTTO + DCE	Hybrid	PD > MO > SC > AD > UA
Spain	Southern Europe	2018	stratified sampling	1000	EQ-VT 1.0	cTTO + DCE	Hybrid	PD > AD > MO > SC > UA
Sweden	Northern Europe	2020	random sampling	785	EQ-VT 2.1	cTTO + DCE	OLS (cTTO)	PD > AD > UA > SC > MO

*Legend: AD = Anxiety and Depression; cTTO = Composite time trade-off; DCE = Discrete Choice Experiment; EQ-VT = EuroQol Valuation Technology; MO = Mobility; OLS = Ordinary Least Squares; PD = Pain and Discomfort; SC = Self-Care; UA = Usual Activities; UNSD = United Nations Statistics Division*

The national value sets generally show globally similar relative importance of each dimension, with some country differences (Table 1). Symptomatic dimensions (PD and AD) were often identified as most important compared to functional ones (MO, UA, and SC). PD was most important in 9 countries (69%), AD in 3 countries (23%), and MO in 1 country (8%). UA was least important in 6 countries (46%), SC in 2 countries (15%), and MO in 1 country (8%). In 7 countries (54%), PD and AD were the top two dimensions, and in 8 countries (62%), the least important dimensions were from the functional group. Four countries (31%) had the exact same ranking: France, Portugal, Poland, and Romania. Analysing the value sets themselves, we found variation among coefficients for level 5 issues across the different dimensions, with Spain having the smallest value for UA (0.018) and Ireland the largest for AD (0.646). The coefficient value range also varied between countries, with Ireland having the largest range (0.65) and Spain the smallest (0.14).

### Cluster analysis of EQ-5D-5L value sets

**Fig. 1:**
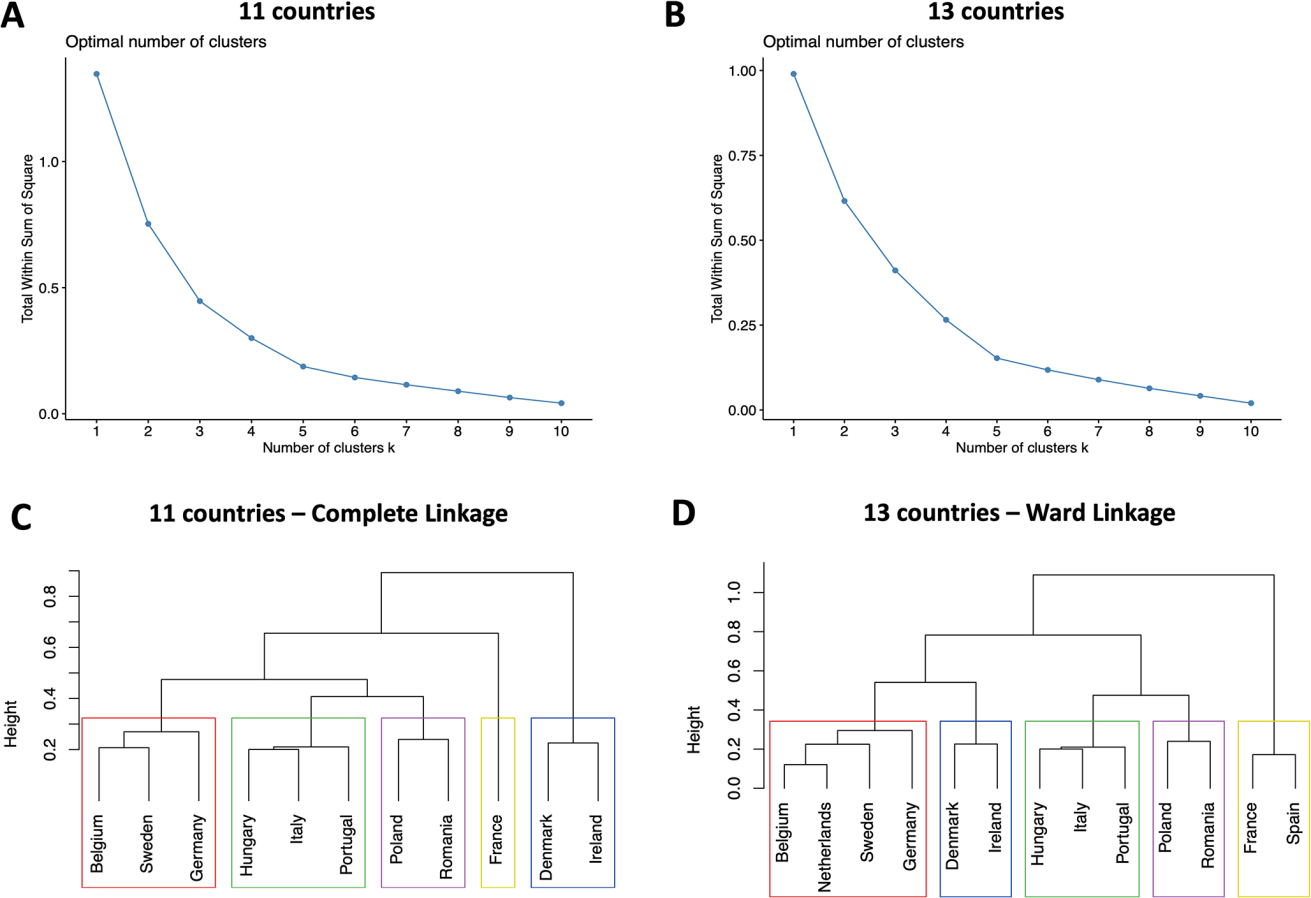
Elbow evaluation method plots (**A** and **B**) and Dendograms (**C** and **D**) for the 11 and 13 country cluster analysis. Panel A: Elbow plot for the 11 country cluster analysis; Panel B: Elbow plot for the 13 country cluster analysis; Panel C: Dendogram for the 11 country cluster analysis; Panel D: Dendogram for the 13 country cluster analysis. NOTE: Coloured rectangles represent similar clusters across panels C and D

The cluster analysis revealed strong structures using Ward linkage in the 13-country (AC = 0.813), and Complete linkage (AC = 0.706) in the 11-country analyses, with Elbow plots suggesting five clusters in both cases. Figure 1 shows the dendograms for these preferred cluster models and Elbow plots.

Results showed minimal differences between the 11 country analysis and 13 country analysis (the inclusion of the two countries (Spain and Netherlands) not using EQ protocols v2.0/2.1): Spain aligns with France in the 13 country analysis (Fig. 1 – panel D), and the Netherlands with Belgium, Sweden and Germany (Fig. 1 – panel D). In both analyses, the Dunn index was computed − 0.78 and 0.83 in the 13 and 11 country analyses, respectively, showing overall moderate to good quality clustering.

**Fig. 2:**
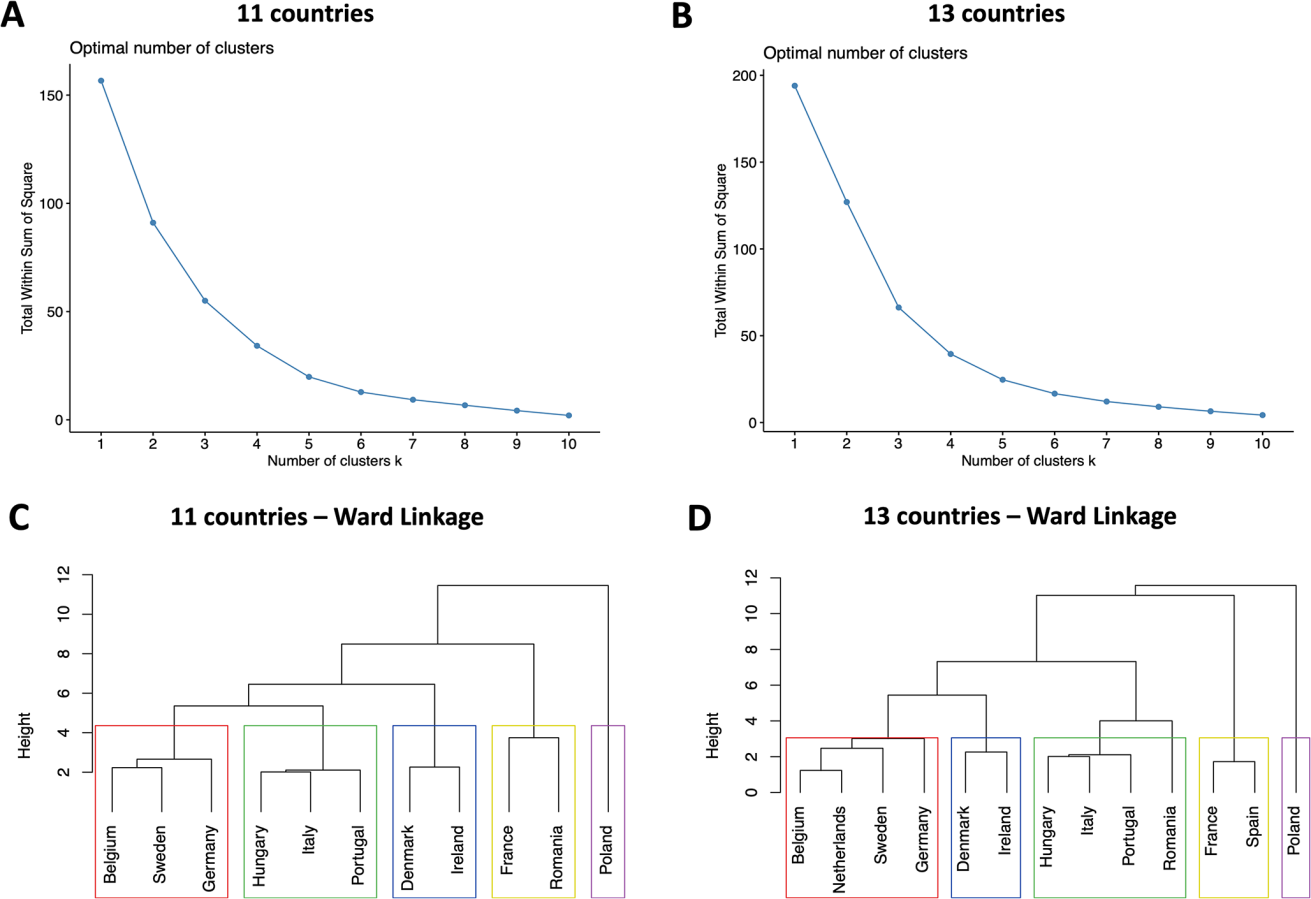
Elbow evaluation method plots (**A** and **B**) and Dendograms (**C** and **D**) for the 11 and 13 country cluster analysis (simulated). Panel A: Elbow plot for the 11 country cluster analysis; Panel B: Elbow plot for the 13 country cluster analysis; Panel C: Dendogram for the 11 country cluster analysis; Panel D: Dendogram for the 13 country cluster analysis. NOTE: Coloured rectangles represent the same clusters across panels C and D

On the second stage, using simulated regression coefficients, Ward linkage is the preferred method in the 11 country (AC = 0.708) and 13 country analysis (AC = 0.749). Elbow plots again suggest an ideal number of 5 clusters. Figure 2 shows the dendograms for these preferred cluster models and respective Elbow plots.

Again, results from the 11 country analysis and 13 country analysis are only slightly different: Fig. 2 – panels C and D). However, the inclusion of these two countries now produced changes in the green cluster, which took in Romania; Poland is alone in the purple cluster in both cases (Fig. 2 – panels C and D). The Dunn index was computed once again − 0.59 and 0.22 in the 13 and 11 country analyses, respectively, showing worse quality clustering than the non-simulated analyses.

Globally, there are no large differences between the non-simulated (Fig. 1) and simulated (Fig. 2) cluster analyses. Belgium, Sweden, Germany and the Netherlands are consistently in the red cluster; Hungary, Italy and Portugal in the green cluster, and Denmark and Ireland in the blue cluster. The purple and, particularly, the yellow clusters were the ones with the largest variations, which suggests these clusters may be absorbing value sets that are less similar between them than the other clusters, making them less well-balanced groups. Taking into account that the non-simulated analyses showed better values for the Dunn Index and the strongest clustering structure (through the AC) was found for the 13 country cluster analysis with the regression coefficients, this is our preferred cluster structure and main estimation.

There is variation in the relative importance of dimensions for each cluster considering both single-dimension level 3 and level 5 issues – Table 2. Considering single-dimension level 5 issues, symptomatic dimensions (PD and AD) are typically rated highest: PD was the most important dimension in all clusters except the blue, which prioritized AD. While PD and AD were the two most important dimensions in the blue and red clusters, PD was followed by MO in the remaining three clusters. UA was the least important dimension in all clusters except the red. All clusters except the yellow (one of two that presented the most variation in country composition) presented the same dimension ranking in all performed analysis. When considering single-dimension level 3 issues the symptomatic dimensions are still the highest rated, but AD is generally more prioritized. AD was the most important dimension in the red, blue and green clusters (in the main analysis) and also in the yellow cluster (in the simulated analysis). UA was mostly considered the least important dimension, as in the level 5 profile, but the ranking of the other dimensions was somewhat different at this severity level. Indeed, while MO was usually the third most important dimension in the severity level 5 profile, in the level 3 profile it was much more commonly last or next to last. While AD was the most important dimension globally, the purple cluster was the only one that placed it last – and it was also the only cluster that placed it next to last in the severity level 5 profile. Finally, the blue cluster was the only one that showed the same dimension ranking in both profiles. This means that preferences generally varied with different severity levels: when considering the most extreme severity level, PD is the most highly valued dimension, while at a moderate severity level, AD takes the top spot, with some variability in the remaining dimensions.

**Table 2:** Relative importance of each dimension in each cluster, based on the comparison of values for health states with single-dimension level 5 issues (51111, 15111, 11511, 11151, 11115) and single-dimension level 3 issues (31111, 13111, 11311, 11131, 11113) across all countries, both in the 11 and 13 country analyses

**SINGLE-DIMENSION LEVEL 5 ISSUES**		
**Coefficient analysis**		
**Cluster**	**Ranking (11 countries)**	Ranking (13 countries)*
Red	**PD** > AD > MO > **UA** > SC	**PD** > AD > MO > **UA** > SC
Blue	AD > **PD** > MO > SC > **UA**	AD > **PD** > MO > SC > **UA**
Green	**PD** > MO > AD > SC > **UA**	**PD** > MO > AD > SC > **UA**
Purple	**PD** > MO > SC > AD > **UA**	**PD** > MO > SC > AD > **UA**
Yellow	**PD** > MO > SC > AD > **UA**	**PD** > MO > AD > SC > **UA**
**Simulated analysis**		
**Cluster**	**Ranking (11 countries)**	**Ranking (13 countries)**
Red	**PD** > AD > MO > **UA** > SC	**PD** > AD > MO > **UA** > SC
Blue	AD > **PD** > MO > SC > **UA**	AD > **PD** > MO > SC > **UA**
Green	**PD** > MO > AD > SC > **UA**	**PD** > MO > AD > SC > **UA**
Purple	**PD** > MO > SC > AD > **UA**	**PD** > MO > SC > AD > **UA**
Yellow	**PD** > MO > SC > **UA** > AD	**PD** > MO > AD > SC > **UA**
**SINGLE-DIMENSION LEVEL 3 ISSUES**		
**Coefficient analysis**		
**Cluster**	**Ranking (11 countries)**	Ranking (13 countries)*
Red	AD > **PD** > SC > **UA** > MO	AD > **PD** > SC > MO > **UA**
Blue	AD > **PD** > MO > SC > **UA**	AD > **PD** > MO > SC > **UA**
Green	AD > MO > **PD** > SC > **UA**	AD > MO > **PD** > SC > **UA**
Purple	**PD** > SC > **UA** > MO > AD	**PD** > SC > **UA** > MO > AD
Yellow	**PD** > AD > MO > SC > **UA**	AD > **PD** > MO > SC > **UA**
**Simulated analysis**		
**Cluster**	**Ranking (11 countries)**	**Ranking (13 countries)**
Red	AD > **PD** > SC > **UA** > MO	AD > **PD** > SC > MO > **UA**
Blue	AD > **PD** > MO > SC > **UA**	AD > **PD** > MO > SC > **UA**
Green	AD > MO > **PD** > SC > **UA**	AD > MO > **PD** > SC > **UA**
Purple	**PD** > SC > **UA** > MO > AD	**PD** > SC > **UA** > MO > AD
Yellow	**PD** > AD > **UA** > MO > SC	AD > **PD** > MO > SC > **UA**

Legend: AD = Anxiety and Depression; MO = Mobility; PD = Pain and Discomfort; SC = Self-Care; UA = Usual Activities. NOTE: PD and UA are in bold as they are most commonly in the extreme positions of the rankings. * marks the preferred cluster structure

**Fig. 3:**
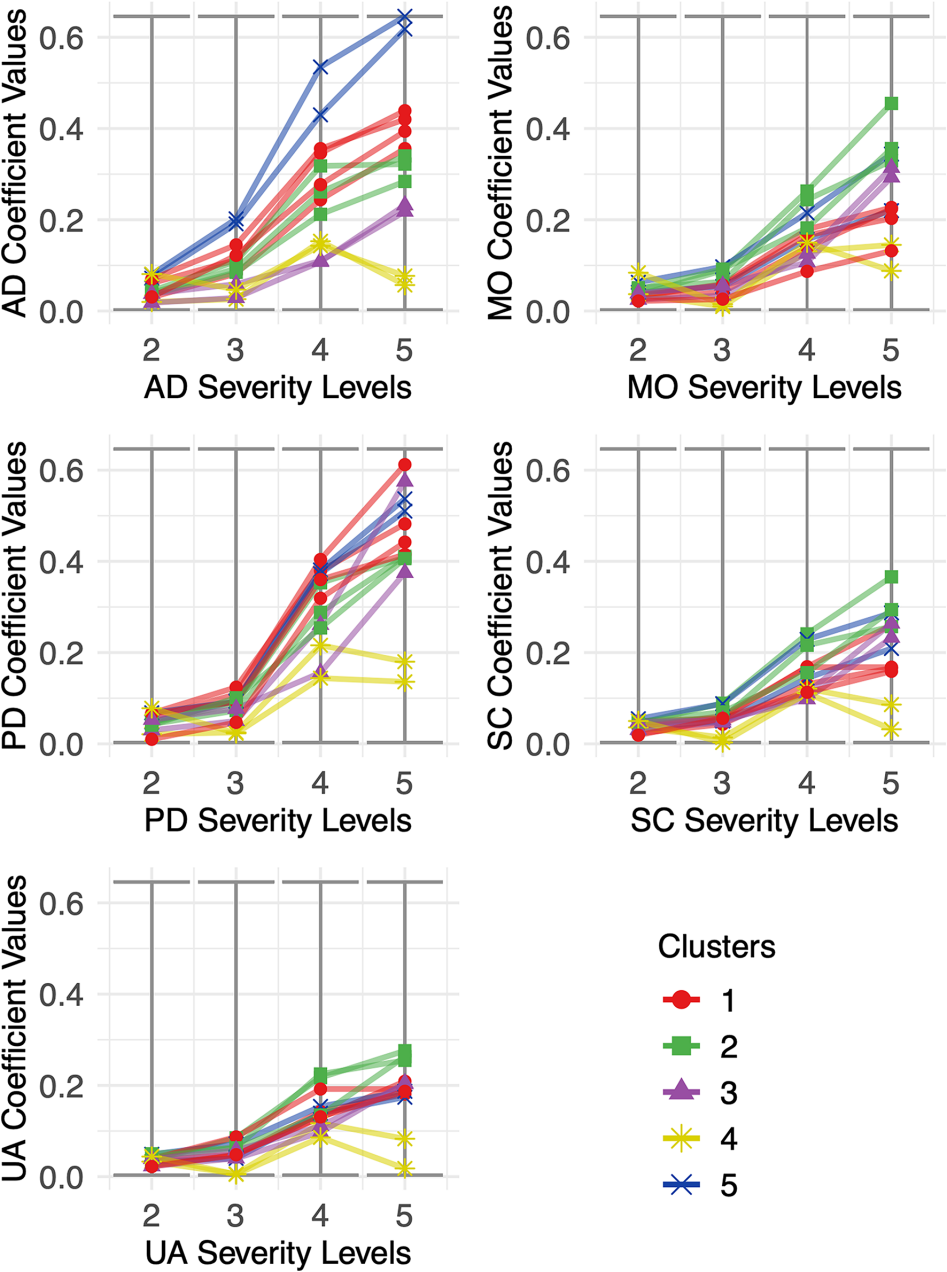
Dimension-level regression coefficient distribution in the included value sets, for each cluster in our main estimation (coefficient-based cluster analysis with 13 countries). Horizontal axis represents severity levels 2–5, relative to severity level 1 (no problems). Vertical axis represents coefficient values for each country (simple mean). Countries in the same cluster are represented by the same color and shape. Cluster 1 (Red) – Belgium, Netherlands, Sweden and Germany. Cluster 2 (Green) – Hungary, Italy and Portugal. Cluster 3 (Purple) - Poland and Romania. Cluster 4 (Yellow) – France and Spain. Cluster 5 (Blue) – Denmark and Ireland. *Legend: AD – Anxiety and Depression. MO – Mobility. PD – Pain and Discomfort. SC – Self-Care. UA – Usual Activities*

Figure 3 presents regression coefficients for levels in each dimension, relative to the reference level (severity level 1 = no problems) for all included value sets. Countries are grouped in clusters across all panels, according to our best cluster definition - results from the main estimation with 13 countries. Each panel represents one of the considered dimensions, and each dot is a country-dimension-level combination. Regression coefficients represent the decrement in health state valuation from moving from level 1 (perfect health) to levels 2 through 5 in each dimension. As expected, countries belonging to the same cluster present similar preferences for each severity dimension-level pair. Globally, PD and AD dimensions present the largest variation in health valuation across severity levels, and the UA dimension the least. All clusters except the yellow one show a clear gradient in health valuation decrement (as expected). However, the yellow cluster shows relatively stable coefficients across all severity levels and dimensions, with a similar trend from levels 2 through 4 in comparison to the other clusters and no further deterioration from level 4 to level 5, in stark contrast to the other clusters.The blue cluster presents the highest coefficients for AD, the green shows higher coefficients for MO, SC and UA.

These results show clear groups considering the value sets and regression coefficients across countries that could not be detected through a simple descriptive analysis or purely geographical divisions – reinforcing the importance of cluster analysis as our chosen method and validating our results.

Sensitivity analysis using K-means clustering yielded identical results – Online Resource 1.

## Discussion

Our findings reveal five distinct clusters of EU-27 countries based on EQ-5D-5L health preferences, which remain robust across sensitivity analyses, including simulated coefficient distributions. This quantitative assessment has significant implications for the transferability of health policies, interventions, or technologies, aiding policymakers in selecting suitable candidate countries. As mentioned earlier, conducting a full EE in each country is resource-intensive and may not always be feasible or necessary at preliminary stages of decision-making. In these cases, our approach provides a cost-effective alternative by allowing countries to reference health policy decisions made by other countries with similar health preference profiles, which can significantly reduce initial costs, offering a pragmatic approach to prioritize or preliminarily assess interventions before investing in a full evaluation. Additionally, clustering based on similar health preferences rather than geographical or cultural proximity enables countries within a cluster to collaborate on health policy and share resources and experiences in a way that may lead to more effective and aligned health policies/interventions.

Our results align with previous research, emphasizing substantial differences in EQ-5D value sets due to methodological and cultural distinctions [11]. As previously mentioned, some studies segmented European countries based on predefined criteria like geography, demography, or politics. Roudijk et al. [8] compared EQ-5D-5 L value sets based on the relative importance of the dimensions, the value scale length and the distribution of values over the scale, and identified three groupings of countries according to preference patterns: Asia, Eastern Europe and Western countries. On the other hand, Łaszewska et al. [12] considered cultural and socioeconomic and demographic factors and found 5 groups of countries, namely English-speaking, Nordic, Central-Western, Southern and Eastern European. The 5 clusters we found in our main estimation are partially in line with these groups: Clusters “**Belgium, Netherlands, Germany and Sweden**” (red), “**France and Spain**” (yellow) and “**Denmark and Ireland**” (blue) would generally correspond to the Western European and Nordic groups, cluster “**Poland and Romania**” (purple) to Eastern Europe. On the other hand, cluster “**Hungary, Italy and Portugal**” (green) is quite uncommon and challenges traditional classifications, suggesting the importance of cultural and socioeconomic factors [30–32].

Consistent patterns in dimension rankings emerged, with symptomatic dimensions (PD and AD) consistently considered more important than functional dimensions (MO, UA and SC). This is line with Roudijk et al.’s findings for Western Europe and, conversely, the opposite pattern for Asian countries. They also found that Eastern European countries were somehow in between - prioritizing PD and MO, with SC, AD and UA following suit. This is partially in line with our results on the relative importance of dimensions for each cluster, as can be seen in the red (Western and Northern European countries) and purple (Eastern European countries) clusters - Table 2. It is interesting to note that both the green cluster (Southern and Central Europe countries) and the yellow cluster (France and Spain; Western and Southern European countries) – placed higher importance to PD and MO dimensions, highlighting the importance of cultural and socioeconomic factors in health preferences of these populations rather than traditional geographical divisions. The blue cluster (Denmark and Ireland) places higher importance to the AD dimension, in line with the literature [33, 34]. It is important to note that Sweden is in a different cluster than Denmark, which can relate to differing perspectives regarding mental health but also differences in access and quality of care [35]. The green cluster (Hungary, Italy and Portugal) has a different pattern, placing higher importance to MO, SC and UA dimensions, which can reflect true differing perspectives [36] or issues regarding access to quality care, urban planning and built environment, which have been documented to impact quality of life in these countries [37, 38].

Our results also showed significant heterogeneity in the importance of PD and AD dimensions across severity levels, both when comparing single dimension level 5 (extreme severity) and level 3 (moderate severity) issues and also when comparing the coefficient in reference to level 1 (no problems), underscoring the influence of cultural, socioeconomic, and healthcare system factors on health preferences. They also suggest a difference in how some countries value moderate vs. severe health problems. In the yellow cluster (France and Spain), the trend from level 2 through 4 seemed aligned with the other clusters, suggesting a similar behaviour to the other countries’ value sets when it comes to the impact of moderate to severe problems. However, the fact that there is no further deterioration from level 4 to level 5 in the yellow cluster, unlike in other groups, suggests that the mean value set derived in this group (and, hence, the original value sets) did not distinguish between the most severe levels of problems in terms of their impact on the overall health state utility score. This in clear contrast with other clusters where there is a noticeable decrease in the utility score when moving from level 4 to level 5. While this could be due to quality of care or societal differences in those countries [39], it could also be attributable to the methodology used in deriving the original value sets themselves, and whether or not it manages to adequately capture these differences.

Roudijk and Łaszewska calculated aggregate value sets for identified groups, suggesting their use for regional decision-making when national sets are unavailable [12]. Unlike studies by Sajjad et al. [11] and Greiner et al. [13], who calculated pan-European value sets, our approach builds on these previous results, highlighting variability within the EU and emphasizing the importance of considering regional differences for successful economic evaluations and health policy/intervention implementation.

Even though ours is the first study of its kind, to the best of our knowledge, one previous work has used cluster analysis to perform transferability assessment. A 2022 study from the OECD [40] identified groups of countries with the greatest potential for successful transfer of a specific intervention based on predefined key success factors. This is an important and interesting perspective, but one which still relies on *a priori* expert considerations to define the clusters, which presents limitations.

Overall, our findings add to the literature on comparing health preferences and transferability of economic evaluations by exploring health preferences of EU-27 countries’ populations and emphasizing the need to consider socioeconomic, demographic, and cultural factors. Recognizing the diversity of perspectives and simultaneously the common visions within the EU is crucial for decision-making and understanding the challenges and opportunities of European integration. Our results can inform the implementation of new health policies, interventions or technologies by identifying target populations for success, while simultaneously fostering closer collaboration between countries within the same cluster. Furthermore, our results also set the scene for future research to address gaps in available value sets in the EU, enabling better-informed policy transfer between jurisdictions and ultimately leading to more equitable health outcomes.

## Strengths & Limitations

The data-driven nature of our study is a clear strength, avoiding pre-assumed groupings that might bias results, allowing for a more realistic understanding of differences and similarities within EU-27 value sets. The hierarchical agglomerative nested clustering method chosen enhances the study’s robustness compared to more common techniques.

One possible limitation of our study is the fact that the full value sets were not always publicly available, which meant regression model coefficients were used instead. However, this is a valid alternative, and clustering based on utility scores derived from full value sets should produce similar results to clustering based on the regression coefficients, given that both are rooted in the same models (linear and non-linear). Still, in order to address possible inaccuracies due to this fact, and despite good overall clustering structure, we conducted sensitivity analysis considering (1) uncertainty, and (2) alternative clustering techniques, like K-means clustering. The simulated coefficient distributions expanded the dataset pool to consider health states coefficients as distributions rather than single points, thus allowing for the inclusion of modelling and distributional uncertainty, and it does not alter the results obtained in the first-stage analysis in an important way. The K-means clustering algorithm assumes a pre-defined number of clusters, which can introduce bias in the process [15]. An additional limitation is the fact that diagnosis tests for determining the optimal number of clusters in hierarchical agglomerative cluster analysis are not consensual. Despite that, the Elbow plot method remains the most commonly used technique to this end, and it consistently pointed to five as the ideal number of clusters. The fact that agglomerative coefficients were generally high, and very high for our preferred cluster structure (AC = 0.813), Dunn index values reflecting moderate separation and compactness of the clusters (0.78 in our preferred cluster structure), and that cluster composition remained globally consistent in all of these analyses are strengths of the study. In fact, these moderate results for the Dunn index may be attributable to the nature of the data used, regression coefficients with relatively low variability and values close to zero. However, data preprocessing techniques such as dimensionality reduction through Principal Component Analysis would be inappropriate as they would lose too much information and make cross-country patterns difficult to assess. Additionally, the other clustering technique tested (K-means) yielded identical results, which reinforces the confidence in our results.

The inclusion of older populations in nationally representative samples raises the issue of population structure composition influencing results, as preferences may differ across age groups. Updating value sets thus becomes crucial with changing population structures, ensuring the relevance of health preferences. It is also important to note that these results are specific to the HRQoL instrument used in the analyses – EQ-5D-5L – and that using another instrument could possibly lead to different results, as they reflect specific conceptual schemes. methodological frameworks and study sample compositions.

Finally, the study’s reliance on only 13 out of 27 EU countries’ value sets is a limitation, stressing the importance for future research to develop more national value sets for comprehensive analysis and decision-making in the EU. Although strategies such as studying the determinants of HRQoL can be a way to overcome missing countries, reapplying cluster analysis when more value sets are available would be useful as a policy tool.

## Conclusion

Despite clear differences in preference patterns across the EU-27, we found five clusters of countries based on the EQ-5D-5L health preferences. These do not directly translate commonly used sub-regional definitions of Europe, suggesting that traditional geographical or political factors may be insufficient to fully capture the cultural values or socioeconomic aspects that contribute to these variety of perspectives on health preferences. These results are essential for the transferability of health policies, interventions or technologies, as they can guide decision makers on which countries could be best candidates, based on EQ-5D-5L value sets. This would be useful for efficient resource allocation in a transfer process in the EU, for the implementation of new interventions, technologies or policies in best-candidate countries and to foster closer collaboration on health policy decisions. While being ready to apply results in the studied countries such as for testing new policies or technologies (i.e., using case studies from different countries), future research is needed to assess national value sets for countries missing this information. In conclusion, EU countries share a common vision and values, but different perspectives and preferences emerge from this study which can inform improved health policies in the EU – thus, fully reflecting the official motto of the EU: “*In varietate concordia*” (“United in diversity”).

## Electronic supplementary material

Below is the link to the electronic supplementary material.

Supplementary Material 1

## Declarations

### Data sharing statement

The data that supports the findings of this study are publicly available in published literature. No restrictions or special licenses were required for the use of these data. Derived data supporting the findings are available from authors upon reasonable request with publication through the corresponding author.

## References

[1] Goeree, R., He, J., O’Reilly, D., et al. (2011). Transferability of health technology assessments and economic evaluations: A systematic review of approaches for assessment and application. *Clinicoecon Outcomes Res*, *3*, 89–104. 21935337 10.2147/CEOR.S14404 PMC3169976

[2] Goeree, R., Burke, N., O’Reilly, D., et al. (2007). Transferability of economic evaluations: Approaches and factors to consider when using results from one geographic area for another. *Current Medical Research and Opinion*, *23*(4), 671–682. 17407623 10.1185/030079906x167327

[3] Devlin, N., Finch, A. P., & Parkin, D. (2022). Guidance to users of EQ-5D-5L value sets. In N. Devlin (Ed.), *Value sets for EQ-5D-5L: A compendium* (pp. 213–233). Springer. Comparative Review & User Guide

[4] Jankovic, D., Bojke, L., Marshall, D., et al. (2021). Systematic Review and Critique of Methods for Economic Evaluation of Digital Mental Health Interventions. *Applied Health Economics and Health Policy*, *19*(1), 17–27. 32803521 10.1007/s40258-020-00607-3

[5] Longworth, L., & Rowen, D. (2013). Mapping to obtain EQ-5D utility values for use in NICE health technologies assessment. *Value in Health*, *16*, 202–210. 23337232 10.1016/j.jval.2012.10.010

[6] National Institute for Health and Care Excellence (2013). Guide to the methods of technology appraisal 2013. Process and methods [PMG 9]. www.nice.org.uk/process/pmg9 27905712

[7] Oppe, M., Rand-Hendriksen, K., Shah, K., et al. (2016). EuroQol protocols for time trade-of valuation of health outcomes. *Pharmacoeconomics*, *34*(10), 993–1004. 27084198 10.1007/s40273-016-0404-1 PMC5023738

[8] Roudijk, B., Janssen, B., & Olsen, J. A. (2022). How Do EQ-5D-5L Value Sets Differ? In N. Devlin, B. Roudijk, K. Ludwig (Eds.), Value Sets for EQ-5D-5L: A Compendium, Comparative Review & User Guide. Springer. Chapter 6. 36810025

[9] Olsen, J. A., et al. (2018). In search of a common currency: A comparison of seven EQ-5D-5L value sets. *Health Economics*, *27*(1), 39–49. 29063633 10.1002/hec.3606

[10] Poudel, N., et al. (2022). Methodological similarities and variations among EQ-5D-5L value set studies: A systematic review. *Journal of Medical Economics*, *25*(1), 571–582. 35416095 10.1080/13696998.2022.2066441

[11] Sajjad, A. (2023). In search of a ‘pan-European value set’; application for EQ-5D-3L. *Bmc Medical Research Methodology*, 23(13). 10.1186/s12874-022-01830-3 PMC9835298 36635625

[12] Łaszewska, A., et al. (2022). Conceptual Framework for Optimised Proxy Value Set Selection through Supra-National Value Set Development for the EQ-5D instruments. *Pharmacoeconomics*, *40*(12), 1221–1234. 36201130 10.1007/s40273-022-01194-y PMC9534733

[13] Greiner, W., et al. (2003). A single European currency for EQ-5D health states. Results from a six-country study. *The European Journal of Health Economics*, *4*(3), 222–231. 15609189 10.1007/s10198-003-0182-5

[14] Knies, S., et al. (2009). Utilities of the EQ-5D: Transferable or not? *Pharmacoeconomics*, *27*(9), 767–779. 19757870 10.2165/11314120-000000000-00000

[15] Everitt, B. S. (2001). Cluster Analysis. 4th Edition, Arnold, London.

[16] EuroQol, W. – In: https://euroqol.org/.

[17] Bouckaert, N. (2022). An EQ-5D-5L Value Set for Belgium. PharmacoEconomics Open, 6: 823–836. 10.1007/s41669-022-00353-3 PMC9362639 35927410

[18] Jensen, C. E., et al. (2021). The Danish EQ-5D-5L Value Set: A hybrid model using cTTO and DCE Data. *Applied Health Economics and Health Policy*, *19*, 579–591. 33527304 10.1007/s40258-021-00639-3 PMC8270796

[19] Andrade, L. F. (2020). A French value set for the EQ-5D-5L. PharmacoEconomics, 38: 413–425. 10.1007/s40273-019-00876-4 PMC7080328 31912325

[20] Ludwig, K., et al. (2018). German value set for the EQ-5D-5L. *Pharmacoeconomics*, *36*, 663–674. 29460066 10.1007/s40273-018-0615-8 PMC5954069

[21] Rencz, F., et al. (2020). Parallel valuation of the EQ-5D-3L and EQ-5D-5L by Time Trade-Off in Hungary. *Value in Health*, *23*(9), 1235–1245. 32940242 10.1016/j.jval.2020.03.019

[22] Hobbins, A., et al. (2018). Utility Values for Health States in Ireland: A Value Set for the EQ-5D-5L. *Pharmacoeconomics*, *36*, 1345–1353. 30051267 10.1007/s40273-018-0690-x PMC6182460

[23] Finch, A. P., et al. (2022). An EQ-5D-5L value set for Italy using videoconferencing interviews and feasibility of a new mode of administration. *Social Science and Medicine*, *292*, 114–519. 10.1016/j.socscimed.2021.114519 34736804

[24] Versteegh, M. F., et al. (2016). Dutch tariff for the five-level version of EQ-5D. *Value In Health : The Journal of the International Society for Pharmacoeconomics and Outcomes Research*, *19*(4), 343–352. 27325326 10.1016/j.jval.2016.01.003

[25] Golicki, D., et al. (2019). Valuation of EQ-5D-5L Health States in Poland: The first EQ-VT-Based study in Central and Eastern Europe. *Pharmacoeconomics*, *37*, 1165–1176. 31161586 10.1007/s40273-019-00811-7 PMC6830402

[26] Ferreira, P. L., et al. (2019). A hybrid modelling approach for eliciting health state preferences: The Portuguese EQ-5D-5L value set. *Quality of Life Research*, *28*, 3163–3175. 31201730 10.1007/s11136-019-02226-5

[27] Olariu, E., et al. (2023). EQ-5D-5L: A value set for Romania. *The European Journal of Health Economics*, *24*, 399–412. 35688994 10.1007/s10198-022-01481-7 PMC10060331

[28] Ramos-Goñi, J. M., et al. (2018). Handling Data Quality issues to Estimate the Spanish EQ-5D-5L value set using a hybrid interval Regression Approach. *Value in Health*, *21*(5), 596–604. 29753358 10.1016/j.jval.2017.10.023

[29] Sun, S. (2022). Estimating a social value set for EQ-5D-5L in Sweden. *Health and Quality of Life Outcomes*, 20(167). 10.1186/s12955-022-02083-w PMC9780618 36564844

[30] Oppe, M., & Van Hout, B. (2017). The power of eliciting EQ-5D-5L values: the experimental design of the EQ-VT EuroQol Working Paper Series, 17003.

[31] Jylhä, M., et al. (1998). Is self-rated health comparable across cultures and genders? The journals of gerontology. *Series B Psychological Sciences and Social Sciences*, *53*(3), 144–152. 10.1093/geronb/53b.3.s144 9602839

[32] Jürges, H. (2007). True health vs response styles: Exploring cross-country differences in self-reported health. *Health Economics*, *16*(2), 163–178. 16941555 10.1002/hec.1134

[33] Anderson, L. M., et al. (2003). Culturally competent healthcare systems. A systematic review. *American Journal of Preventive Medicine*, *24*(3 Suppl), 68–79. 12668199 10.1016/s0749-3797(02)00657-8

[34] Ventegodt, S., et al. (2008). Which factors determine our quality of life, health and ability? Results from a Danish population sample and the Copenhagen perinatal cohort. *J Coll Physicians Surg Pak*, *18*(7), 445–450. 18760073

[35] Conry, M. C., et al. (2011). The clustering of health behaviours in Ireland and their relationship with mental health, self-rated health and quality of life. *BMC Public Health*, *11*, 692. 21896196 10.1186/1471-2458-11-692 PMC3187756

[36] OECD. (2021). Accessible, high-quality mental health services. *A New Benchmark for Mental Health systems: Tackling the social and economic costs of Mental Ill-Health*. OECD Publishing.

[37] Péntek, M., et al. (2020). Acceptable health and ageing: Results of a cross-sectional study from Hungary. *Health and Quality of life Outcomes*, *18*(1), 346. 33081803 10.1186/s12955-020-01568-w PMC7574437

[38] Paiva, M. M., & Villarouco, V. (2021). Acessibility in collective housing for the elderly: A case study in Portugal. *Work (Reading Mass)*, *41*(Suppl 1), 4174–4179. 10.3233/WOR-2012-0716-4174 22317362

[39] Álvarez-Gálvez, J. (2022). Social inequalities in multimorbidity patterns in Europe: A multilevel latent class analysis using the European Social Survey (ESS). SSM - population health, 20, 101–268. 10.1016/j.ssmph.2022.101268 PMC9638822 36353098

[40] Wiper, O. (2022). Cluster analysis to assess the transferability of public health interventions, OECD Health Working Papers, No. 133, OECD Publishing, Paris.

