# Supplementary material for: In varietate concordia – cluster analysis of EQ-5D-5L value sets in European Union countries
Source: Qual Life Res. 2024 Dec 12;34(4):1091–102. doi: 10.1007/s11136-024-03872-0 (PMC11982070; doi:10.1007/s11136-024-03872-0)
Supplement: Supplementary file 1 — Supplementary Material 1 [file 11136_2024_3872_MOESM1_ESM.pdf]

SUPPLEMENTARY MATERIAL

"In varietate concordia – Cluster Analysis of EQ-5D-5L value sets in European Union countries" – Quality of Life Research

Authors: Vera Pinheiro, Tallys Feldens, Juanita A. Haagsma, João Vasco Santos.
Corresponding author: Vera Pinheiro - Public Health Unit, Matosinhos Local Health Unit, Matosinhos, Portugal; CINTESIS, Centre for Health Technology and Services Research, Porto, Portugal; MEDCIDS, Faculty of Medicine, University of Porto, Portugal
Email: verafcpinheiro@gmail.com

K-Means Clustering

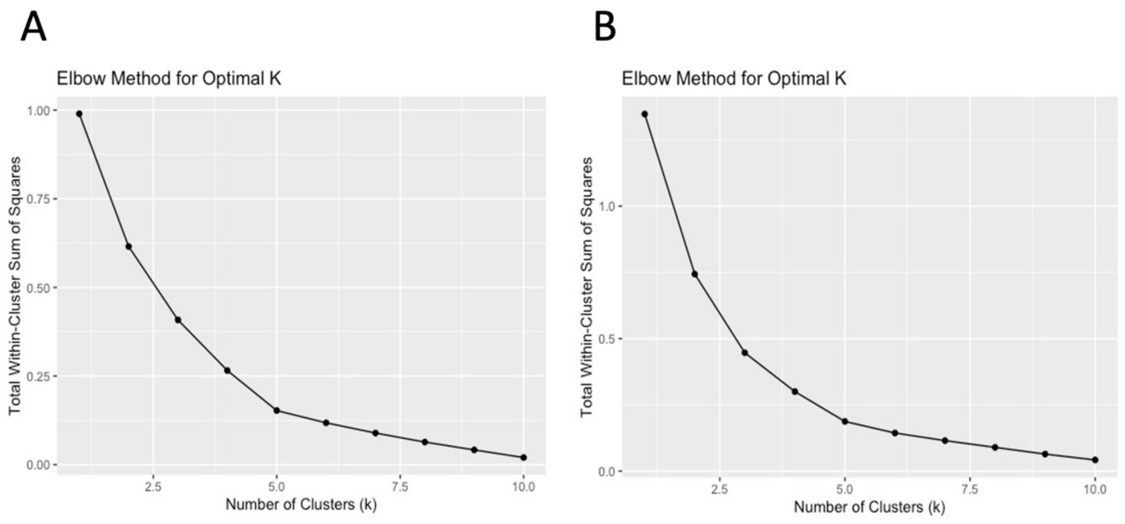

Figure 1. Elbow evaluation method plots for the 11 (A) and 13 (B) country cluster analysis with K-Means.

Optimal number of clusters (k) = 5

- 11 country analysis

Cluster 1	Hungary, Italy, Portugal
Cluster 2	Romania, Poland
Cluster 3	Denmark, Ireland
Cluster 4	France
Cluster 5	Belgium, Germany, Sweden

Compactness of clusters - Within cluster sum of squares by cluster:
(lower values mean more compact clusters)

1- 0.04221133
2- 0.02874450
3- 0.05614467
4- 0.02554400
5- 0.00000000

Separation of clusters (between_SS / total_SS):
(higher ratio means more separated clusters)

84.6 %

Conclusion: Clusters are also likely well defined and meaningful.

- 13 country analysis

Cluster 1	Belgium, Germany, Netherlands, Sweden
Cluster 2	Romania, Poland
Cluster 3	Hungary, Italy, Portugal
Cluster 4	Denmark, Ireland
Cluster 5	France, Spain

Compactness of clusters - Within cluster sum of squares by cluster:
(lower values mean more compact clusters)

1- 0.07614500
2- 0.02874450
3- 0.04221133
4- 0.02554400
5- 0.01476000

Separation of clusters (between_SS / total_SS):
(higher ratio means more separated clusters)

86.1 %

Conclusion: Clusters are likely well defined and meaningful.
